# Percepciones de los médicos residentes sobre el desarrollo del programa de residentado de cardiología durante la pandemia por COVID-19 en Lima, Perú

**DOI:** 10.47487/apcyccv.v3i3.230

**Published:** 2022-09-30

**Authors:** Adriel Olortegui Yzu, Rosalía Fernández Coronado

**Affiliations:** 1 Departamento de Medicina Preventiva y Salud Pública, Facultad de Medicina, Universidad Nacional Mayor de San Marcos. Lima, Perú Universidad Nacional Mayor de San Marcos Departamento de Medicina Preventiva y Salud Pública Facultad de Medicina Universidad Nacional Mayor de San Marcos Lima Peru; 2 Facultad de Medicina, Universidad Nacional Mayor de San Marcos. Lima, Perú Universidad Nacional Mayor de San Marcos Facultad de Medicina Universidad Nacional Mayor de San Marcos Lima Peru

**Keywords:** Internado y Residencia, Cardiología, Especialización, Infecciones por Coronavirus, Perú, Internship and Residency, Cardiology, Specialization, Coronavirus Infections, Peru

## Abstract

**Objetivo:**

. Describir las percepciones de los médicos residentes acerca del desarrollo de su programa de formación durante la pandemia de la COVID-19 en la ciudad de Lima - Perú.

**Materiales y métodos:**

. Mediante un estudio transversal se aplicó un cuestionario a 78 residentes de cardiología de los dos últimos años de formación en la especialidad. Se evaluó las percepciones sobre el acompañamiento y soporte de las universidades en las sedes docentes, para el desarrollo del programa de formación en cardiología durante la pandemia de la COVID-19.

**Resultados:**

. Con relación al apoyo brindado para su formación, los rubros evaluados mostraron deficiencias por encima del 60%, donde la supervisión permanente faltó en el 90,0% de los residentes. Sobre el cumplimiento de las rotaciones, los residentes solo recibieron supervisión en el 24,4%, observándose que no lograron realizar rotaciones adecuadas en el 80,8% de los casos. Los cursos del plan curricular fueron adecuadamente desarrollados en el 92,5% de los casos y las acciones por la salud del residente fueron muy bajas, destacando que solo en el 9,0% de los residentes la universidad indagó acerca del estado de salud del residente.

**Conclusiones:**

. El desarrollo del programa de formación del residentado de cardiología durante la pandemia de la COVID-19, presentó falencias importantes, evidenciándose que se acentuaron las deficiencias en comparación a estudios previos. Es importante evaluar los niveles y características del desarrollo de los programas de residentado médico una vez que la pandemia se aleje.

## Abstract (en)

**Objective.:**

To describe the perceptions of resident doctors about the development of their training program during the pandemic in the city of Lima - Peru.

**Materials and methods.:**

Through a cross-sectional study, a questionnaire was applied to seventy-eight cardiology residents in the last two years of training in the specialty. The perceptions about the accompaniment and support of the universities in the educational venues, for the development of the training program in cardiology during the pandemic, were evaluated.

**Results.:**

Regarding the support provided for their training, the items evaluated showed shortcomings above 60%, where permanent supervision was lacking in 90.0% of the residents. Regarding compliance with the rotations, the residents only received supervision in 24.4%, observing that they did not manage to carry out adequate rotations in 80.8% of the cases. The courses of the curricular plan were adequately developed in 92.5% of the cases, and the actions for the health of the resident were very low, highlighting that only in 9.0% of the cases did the university inquire about the state of health of the resident.

**Conclusions.:**

The development of the cardiology residency training program during the pandemic presented important shortcomings, showing that the deficiencies were accentuated compared to previous studies.

## INTRODUCCIÓN

La formación de médicos especialistas en el Perú está enmarcada dentro del Sistema Nacional de Residentado Médico (SINAREME), cuya estructura, componentes, funcionamiento y financiamiento, está establecido por ley. Dicha norma establece que el sistema está integrado por el Ministerio de Salud (MINSA), por universidades que cuentan con programas de segunda especialización en medicina, las instituciones prestadoras de servicios de salud (IPRESS), gobiernos regionales, Colegio Médico del Perú y la Asociación Nacional de Médicos Residentes ^1^^,^^2^.

El SINAREME establece su estructura a partir de las funciones de sus componentes, las universidades se encargan de las actividades formativas, los establecimientos de salud proveen el campo clínico y el MINSA es el rector del sistema. A partir de estos componentes, se establecen los órganos de gestión, que tienen tres niveles: el Consejo Nacional de Residentado Médico (CONAREME) a nivel nacional, el Consejo Regional de Residentado Médico (CORERE) a nivel regional y el Comité de Sede Docente. En cada uno de estos niveles están representados los componentes establecidos del SINAREME ^1^^,^^3^; es decir, hay representantes del MINSA o Gobiernos regionales, de los establecimientos, de las universidades y de la sede docente de los residentes, según corresponda.

Los mecanismos de integración e interacción de los actores, establecen un marco normativo que integra los establecimientos de salud con las universidades y las condiciones de colaboración, que van desde la adscripción de convenios de prestaciones y contraprestaciones, hasta la definición de los recursos humanos y logísticos que debe contar un campo clínico que funcione en una sede docente acreditada. Este contexto incluye, el soporte formativo, laboral y de formación académica, científica y profesional que deben asegurar de manera conjunta ^4^^-^^6^. Esta alianza busca asegurar que los establecimientos se comporten como hospitales o clínicas docentes, que es lo que sustenta el funcionamiento de este tipo de programas en otros países ^7^^,^^8^.

En función de todo lo anterior, las universidades deben asegurar que las sedes docentes cumplan lo siguiente: i) apoyo en los procesos de formación; ii) supervisión de los procesos formativos y cumplimiento curricular; iii) desarrollo de cursos del plan curricular y, iv) velar por la salud del residente ^1^^,^^4^^,^^6^.

La pandemia por la COVID-19, obligó el confinamiento de los trabajadores e implantó la reconversión hospitalaria, estas medidas alteraron la dinámica de trabajo entre las universidades y los establecimientos de salud, lo que influyó en la relación académica y formativa que los residentes mantienen con sus universidades y sedes docentes ^9^^,^^10^.

Diversos estudios han mostrado la afectación de la formación de los programas de segunda especialización en medicina, principalmente desde la perspectiva de la reducción del volumen, siendo limitados los estudios que lo hayan abordado desde la perspectiva de los residentes e incluyendo el rol de las universidades en su ámbito formativo ^11^^-^^14^; por lo que el objetivo del presente estudio es caracterizar las percepciones sobre el soporte formativo y apoyo brindado a los residentes de cardiología de la ciudad de Lima por las universidades y las sedes docentes durante la pandemia de la COVID-19. La finalidad es conocer desde la perspectiva del médico residente, las características del efecto sobre su formación de un contexto de pandemia a fin de mitigar los efectos negativos de futuras situaciones de emergencia sanitaria.

## MATERIALES Y MÉTODOS

### Diseño del estudio

Se realizó una encuesta en el mes de julio de 2021 a los médicos residentes que cursaban los dos últimos años de todos los programas de residentado en la especialidad de cardiología en la ciudad de Lima. Cabe precisar que en el caso del programa que conduce la Universidad Peruana Cayetano Heredia, se incluyeron a los residentes de tercer y cuarto año, debido a que el programa de residentado dura cuatro años en dicha universidad ^15^, considerándolos para los fines de análisis como residentes de segundo y tercer año respectivamente.

En el estudio fueron incluidos los 95 residentes de cardiología que cursaron los dos últimos años del programa en el periodo de pandemia por la COVID-19 comprendido entre julio de 2020 y junio de 2021 ^16^^,^^17^.

### Variables del estudio

Se diseñó un cuestionario de 22 preguntas las cuales indagaban sobre aspectos personales, sede docente, año del residentado, universidad del programa de residentado, nivel de pertinencia para la formación del residente por el establecimiento de salud y las percepciones sobre el soporte e interacción con su universidad. La legibilidad del instrumento fue validada mediante la aplicación del cuestionario a seis residentes de otras especialidades del instituto, quienes manifestaron que el instrumento era claro y entendible y sin redundancias. Las variables de percepción sobre la interacción con la universidad y formación en la sede docente con el residente se muestran en la Tabla 1.

**Tabla 1:** Variables de percepción sobre el soporte formativo y apoyo del programa de residentado en cardiología

Variables incluidas en el estudio
Apoyo procesos de formación
- Soporte para mantener el nivel de formación (a)
- Apoyo óptimo a la formación durante la pandemia (a)
- Supervisión permanente del cumplimiento cabal de su formación (c)
Supervisión y cumplimiento rotaciones
- Supervisión del cumplimiento de las rotaciones durante la pandemia (a)
- Realización de rotaciones externas que han reforzado su formación como cardiólogo (c)
- La pandemia afectó su formación (c)
- La sede cubrió las expectativas de formación del residente (c)
Desarrollo de los cursos del Plan
- Desarrollo de la totalidad de cursos del plan curricular (c)
- Puntualidad para el desarrollo de los cursos (a)
- Medios para desarrollo de los cursos:
Uso de material impreso (a)
Clases por vídeos (a)
Clases sincrónicas (a)
Clases asincrónicas (a)
- Grado de estructuración de los cursos (a)
Atención a la salud del médico residente
- Capacitación sobre aspectos preventivos de la COVID-19 (a)
- Conocimiento del estado de vacunación del residente (b)
- Capacitación o estrategias para proteger su salud mental (b)
- Indagación sobre el estado de salud del médico residente

a. Respuesta dicotómica.

b. Respuesta de tres opciones.

c. Respuestas con escala de Likert.

La mayoría de las variables de interacción con la universidad fueron medidas mediante preguntas dicotómicas (Sí/No - ítems con letra «a» en la Tabla 1). Las variables de desarrollo de los cursos del plan curricular, protección de la salud mental y conocimiento del estado de vacunación del residente fueron politómicas, siendo estas enfocadas al cumplimiento o incumplimiento del ítem o la no correspondencia o desconocimiento de la ejecución de la variable por parte del residente (letra «b» en la Tabla 1). Las variables restantes, principalmente las de formación en la sede docente, fueron recopiladas mediante una escala de Likert acerca del acuerdo con el desarrollo de la variable (letra «c» en la Tabla 1).

El cuestionario se elaboró en Microsoft Forms® y distribuido individualmente utilizando la red social WhatsApp®, reiterándose los mensajes de participación hasta en cuatro oportunidades a los residentes que no respondían. Para evitar la duplicación de respuestas y realizar el seguimiento de los respondientes, se recopiló el correo electrónico de los residentes, que constituyó el único dato personal que se recopiló, no siendo utilizado para ningún otro fin que el de seguimiento y evitar la duplicación de respuestas.

### Análisis estadístico

Las respuestas fueron exportadas a una hoja de cálculo de Microsoft Excel®, para realizar el control de calidad de las respuestas, así como la depuración correspondiente. Para el análisis estadístico, los registros fueron exportados al formato de archivo de IBM SPSS Statistics Versión 24.0 (Armonk, NY: IBM Corp.).

El análisis estadístico consistió en la obtención de las medidas de resumen y frecuencias de todas las variables. A partir de los resultados descriptivos se caracterizó el comportamiento de las variables, específicamente el tipo de universidad y de organización de la sede docente; y, su asociación con las variables de interacción con la universidad y formación en la sede docente. Estas asociaciones fueron sometidas a prueba de hipótesis estadística de chi cuadrado o Fisher con fines exploratorios.

### Aspectos éticos

Este estudio fue aprobado por el Comité de Ética del Instituto Nacional Cardiovascular «Carlos Alberto Peschiera Carrillo» de la Seguridad Social del Perú, mediante Certificado de Aprobación 24/2021-CEI del 28 de junio de 2021.

## RESULTADOS

### Características generales

Ochenta residentes accedieron a la encuesta, obteniéndose 78 respuestas válidas (1 declinó responder la encuesta y 1 registro tuvo una proporción importante de preguntas que no fueron respondidas). La cobertura de la encuesta fue de 82,1%. El 57,7% (45 encuestados) fueron residentes de segundo año. Con relación a las características personales, el 78,2% correspondió al sexo masculino. La media de edad fue de 32,3 años. En su mayoría, un 62,8% (49 residentes) fueron en universidades públicas. El tipo de organización sanitaria de la sede docente más frecuente fue el MINSA y la Seguridad Social del Perú (EsSalud) con 43,6%. La incidencia de COVID-19 entre los residentes encuestados fue de 29,5% de residentes y el trabajo de atención de pacientes en área COVID-19 tuvo que ser cumplido por el 76,9% de los residentes de cardiología.

### Apoyo en procesos de formación

Los residentes manifestaron que la universidad brindó apoyo suficiente para mantener su nivel de formación en el 33,3% de los casos, proporción mayor en las universidades privadas con relación a las públicas (37,9 y 30,6%, respectivamente). En el caso del apoyo óptimo de la universidad para su proceso de formación durante la pandemia por la COVID-19, el 15,4% de los residentes manifestó haber recibido apoyo, con ligero predominio para las universidades privadas (17,2% versus 14,3%, respectivamente). Acerca de la supervisión permanente para lograr una formación a cabalidad, solo el 9,0% lo recibió, un 14,3% de los residentes de las universidades públicas indico estar «de acuerdo» haberla recibido, en comparación con las universidades públicas los cuales ninguno recibió dicha formación. Los resultados de este acápite se encuentran en la Tabla 2.

**Tabla 2:** Apoyo en los procesos de formación brindados al residente por la universidad durante la pandemia de la COVID-19

Apoyo procesos de formación	Tipo de universidad				Total		Valor p^a^
	Privada		Pública							
	n (29)	%	n (49)	%	n (78)	%		
La universidad le ha brindado apoyo académico para mantener su nivel de formación							
Sí	11	37,9	15	30,6	26	33,3	0,337
No	18	62,1	34	69,4	52	66,7	
La universidad ha apoyado de manera óptima mi formación en el residentado							
Totalmente en desacuerdo	4	13,8	12	24,5	16	20,5	0,492
En desacuerdo	11	37,9	12	24,5	23	29,5				
Ni de acuerdo ni en desacuerdo	9	31,0	18	36,7	27	34,6				
De acuerdo	5	17,2	7	14,3	12	15,4		
Mi universidad supervisó permanentemente el cumplimiento cabal de mi formación							
Totalmente en desacuerdo	8	27,6	19	38,8	27	34,6	0,103
En desacuerdo	17	58,6	16	32,7	33	42,3				
Ni de acuerdo ni en desacuerdo	4	13,8	7	14,3	11	14,1				
De acuerdo	0	0,0	5	10,2	5	6,4				
Totalmente de acuerdo	0	0,0	2	4,1	2	2,6			

a: prueba de chi cuadrado o Fisher.

### Supervisión y cumplimiento de rotaciones

Con relación a la supervisión del cumplimiento de las rotaciones durante la pandemia por la COVID-19, solo el 24,4% consideró que la universidad supervisó este aspecto, proporción más alta en las universidades privadas (31,0%) que en las públicas (20,4%). En lo que respecta a la realización de rotaciones externas que han reforzado la formación como cardiólogo, el 75,6% se mostraron desacuerdo al respecto, las universidades privadas mostraron una mayor proporción (79,6%) en con relación a las universidades públicas (69,0%). Sobre la capacidad de la sede docente para cubrir las necesidades de formación, el 51,3% se mostró en desacuerdo y solamente el 21,2% mostró estar de acuerdo. La proporción de desacuerdo fue mayor en las universidades públicas (55,1%) con relación a las privadas (44,8%). Asimismo, un 83,3% de los residentes indicó que su formación se vio afectada por la pandemia de la COVID-19, siendo esta percepción mayor en los residentes de las universidades públicas (89,8%) en comparación con las privadas (72,4%). Estos resultados pueden verse con más detalle en la Tabla 3.

**Tabla 3:** Percepción sobre la supervisión y cumplimiento de las rotaciones provistas a los médicos residentes por la universidad durante la pandemia de la COVID-19

Apoyo procesos de formación	Tipo de universidad				Total		Valor p^a^
	Privada		Pública						
	n (29)	%	n (49)	%	n (78)	%		
Supervisión del cumplimiento de su plan de rotaciones			
No	20	69,0	39	79,6	59	75,6	0,291
Sí	9	31,0	10	20,4	19	24,4	
He podido realizar rotaciones externas que han reforzado mi formación como cardiólogo			
Totalmente en desacuerdo	10	34,5	21	42,9	31	39,7	0,217
En desacuerdo	10	34,5	18	36,7	28	35,9	
Ni de acuerdo ni en desacuerdo	2	6,9	2	4,1	4	5,1	
De acuerdo	4	13,8	8	16,3	12	15,4	
Totalmente de acuerdo	3	10,3	0	0,0	3	3,8	
Mi sede logró cubrir mis necesidades de formación durante la pandemia			
Totalmente en desacuerdo	2	6,9	11	22,4	13	16,7	0,308
En desacuerdo	11	37,9	16	32,7	27	34,6	
Ni de acuerdo ni en desacuerdo	5	17,2	11	22,4	16	20,5	
De acuerdo	8	27,6	7	14,3	15	19,2	
Totalmente de acuerdo	3	10,3	4	8,2	7	9,0	
Mi formación se ha visto afectada por la pandemia			
Totalmente en desacuerdo	0	0,0	2	4,1	2	2,6	0,060
En desacuerdo	4	13,8	2	4,1	6	7,7	
Ni de acuerdo ni en desacuerdo	4	13,8	1	2	5	6,4	
De acuerdo	9	31,0	13	26,5	22	28,2	
Totalmente	12	41,4	31	63,3	43	55,1	

a: prueba de chi cuadrado o Fisher.

### Desarrollo de los cursos del plan curricular

Con relación al cumplimiento de los cursos del plan curricular, 38 residentes (48,7%) indicaron que no les correspondía realizar cursos, por lo que los resultados sobre el cumplimiento de los cursos se presentan sobre un total de 40 residentes que respondieron las preguntas relacionadas a este conjunto de variables. En este sentido, el 92,5% de los residentes indicaron que realizaron la totalidad de sus cursos, observándose una mayor proporción de cumplimiento en las universidades públicas con relación a las privadas (96,3 y 84,6%, respectivamente). La puntualidad de desarrollo de los cursos se cumplió en el 52,5% de los casos, igualmente con mayor cumplimiento para las universidades públicas (59,3%) con relación a las privadas (38,5%). El 77,5% considero que se desarrolló la totalidad de los contenidos de los cursos, siendo similar la proporción de las universidades privadas con las públicas (76,9% versus 77,8%).

En lo que respecta a los medios utilizados para el desarrollo de los cursos, solo el 7,5% usó material impreso, correspondiendo la totalidad a las universidades públicas. El 72,5% de los residentes indicaron que desarrollaron clases mediante videos por internet, evidenciándose predominio por parte de las universidades privadas. Con relación al momento de su realización, las clases fueron sincrónicas en el 87,5%, predominando en las universidades públicas (96,3%) frente a las privadas (69,2%), diferencia que resultó estadísticamente significativa. Los cursos con contenido asincrónico se realizaron en el 40,0% de los casos, con predominio a favor de las universidades privadas con relación a las públicas (46,2 y 37,0%, respectivamente). El 65,0% de los residentes manifestaron que los cursos estuvieron completamente estructurados, siendo el predominio de las universidades públicas con 81,5%, mientras que, en las privadas, solo lo fue en el 30,8%, esta diferencia fue estadísticamente significativa. La distribución sobre estos aspectos del desarrollo de los cursos puede verse en la Tabla 4.

**Tabla 4:** Percepción sobre el desarrollo de los cursos del Plan curricular por la universidad durante la pandemia de la COVID-19

Apoyo procesos de formación	Tipo de universidad				Total		Valor p^a^
	Privada		Pública						
	n (13)	%	n (27)	%	n (40)	%		
Desarrollo de la totalidad de cursos del Plan Curricular							
Sí	11	84,6	26	96,3	37	92,5	0,242
No	2	15,4	1	3,7	3	7,5	
Cursos desarrollados puntualmente							
Sí	5	38,5	16	59,3	21	52,5	0,185
No	8	61,5	11	40,7	19	47,5	
Contenidos de los cursos desarrollados totalmente							
Sí	10	76,9	21	77,8	31	77,5	0,624
No	3	23,1	6	22,2	9	22,5	
Curso desarrollado con material impreso							
Sí	0	0,0	3	11,1	3	7,5	0,296
No	13	100,0	24	88,9	37	92,5	
Curso desarrollado con vídeos por internet							
Sí	10	76,9	19	70,4	29	72,5	0,486
No	3	23,1	8	29,6	11	27,5	
Clases desarrolladas de manera sincrónica							
Sí	9	69,2	26	96,3	35	87,5	0,031
No	4	30,8	1	3,7	5	12,5	
Clases virtuales asincrónicas							
Sí	6	46,2	10	37,0	16	40,0	0,415
No	7	53,8	17	63,0	24	60,0	
Los cursos estuvieron completamente estructurados							
Sí	4	30,8	22	81,5	26	65,0	0,003
No	9	69,2	5	18,5	14	35,0	

a: prueba de chi cuadrado o Fisher.

### Atención a la salud del residente

El 16,7% de los residentes recibió capacitación sobre aspectos preventivos de la COVID-19 por parte de la universidad, siendo las universidades privadas las que lo hicieron en una proporción mayor que las públicas, diferencia que resultó estadísticamente significativa (Tabla 5). El conocimiento del estado de vacunación del residente solo fue indagado por parte de la universidad en el 6,4% de los residentes. El desarrollo de capacitación sobre la salud mental del residente fue indagado en el 21,8% de los residentes por parte de la universidad, mostrando una mayor proporción las universidades privadas que las públicas.

**Tabla 5:** Acciones por la salud del médico residente realizadas por la universidad durante la pandemia de la COVID-19

Apoyo procesos de formación	Tipo de universidad				Total		Valor p^a^
	Privada		Pública							
	n (29)	%	n (49)	%	n (78)	%	
Capacitación sobre aspectos preventivos de la COVID-19	8	27,6	5	12,8	13	16,7	0,047
Mecanismo para conocer el estado de vacunación del residente por parte de la universidad					
No	20	69,0	38	77,6	58	74,4	0,679
No sabe	7	24,1	8	16,3	15	19,2	
Sí	2	6,9	3	6,1	5	6,4	
Capacitación o estrategias para proteger su salud mental por parte de la universidad					
No	18	62,1	33	67,3	51	65,4	0,604
No sabe	3	10,3	7	14,3	10	12,8	
Sí	8	27,6	9	18,4	17	21,8	
Mi universidad se mantuvo al tanto de mi estado de salud					
Totalmente en desacuerdo	11	37,9	29	59,2	40	51,3	0,233
En desacuerdo	12	41,4	13	26,5	25	32,1	
Ni de acuerdo ni en desacuerdo	3	10,3	3	6,1	6	7,7	
De acuerdo	3	10,3	2	4,1	5	6,4	
Totalmente de acuerdo	0	0,0	2	4,1	2	2,6	

a: prueba de chi cuadrado o Fisher.

## DISCUSIÓN

El efecto de la pandemia por la COVID-19 en la formación de médicos residentes ha ocasionado una menor calidad de la formación, asociada principalmente a un menor volumen de participación o realización en actividades asistenciales de la especialidad, describiéndose mayor afectación en las especialidades quirúrgicas e intervencionistas ^18^. Se ha determinado la ocurrencia de una menor adquisición de competencias clínicas por la disminución de rotaciones de formación ^13^, una reducción de rotaciones externas a la sede docente para reforzar la formación, la disminución de la cantidad de procedimientos quirúrgicos y, en el caso específico de la formación en cardiología, menor número de procedimientos intervencionistas así como de rotaciones para reforzar la formación en la especialidad ^12^^,^^19^^,^^20^. En el caso específico del Perú, la formación de especialistas requiere del trabajo coordinado y estrecho de los estamentos definidos en la Ley del SINAREME ^1^, por lo que se esperaría que durante la pandemia por la COVID-19, el trabajo coordinado de estos, compensaría los efectos negativos que esta ha generado en la formación de los médicos especialistas del Perú.

El presente estudio explora estos aspectos desde una perspectiva descriptiva y, específicamente, para la especialidad de cardiología; con la finalidad de conocer cómo ha afectado la pandemia por la COVID-19 la formación de cardiólogos en el Perú. Nuestros resultados muestran una serie de limitaciones en la interrelación entre la institución formadora, sede docente hospitalaria y médico residente de cardiología, lo que estaría asociado a la heterogeneidad que cada sede docente y universidad tiene para sus procesos de formación y evaluación del residente ^21^, además de los cambios en la dinámica del trabajo asistencial que se implantó en las instituciones formadoras y hospitales como resultado de la pandemia ^9^.

Este estudio muestra que, durante la pandemia, las actividades de apoyo a la formación brindadas por la universidad a los residentes, tuvieron falencias. En 3 de cada 4 residentes, la universidad no cumplió con supervisar el cumplimiento de las rotaciones, con una variación a favor de las universidades privadas (2 de cada 3) frente a las públicas (4 de cada 5). Asimismo, 7 de cada 8 residentes no recibió apoyo para alcanzar una formación óptima y 9 de cada 10 no recibió supervisión del cumplimiento cabal de sus rotaciones, resultados que se condicen con la elevada proporción de residentes que no recibió apoyo para mantener un nivel cabal de formación durante la pandemia (9 de cada 10 diez). Estos resultados muestran que la pandemia redujo la calidad de la formación, comparando con el 48,2% de residentes que declaró tener buena calidad de formación y con el 77,2% que tuvo supervisión de su tutor en un estudio previamente realizado en hospitales públicos de Lima y el Callao ^22^.

Con relación al cumplimiento de las rotaciones del plan curricular y la supervisión correspondiente que deben realizar las instituciones formadoras, también se evidencian falencias marcadas: 3 de cada 4 residentes manifestaron no haber tenido supervisión para el cumplimiento de su plan de rotaciones. Asimismo, 7 de cada 8 residentes indicaron que no lograron realizar rotaciones externas que reforzaran su formación, aunque 1 de cada 3 manifestó que su sede logró cubrir sus expectativas de formación. Finalmente, 9 de cada 10 residentes consignaron que la pandemia afectó su formación como residentes de cardiología. Estos aspectos han sido abordados en otros estudios, Miní ^22^ por ejemplo, halló que el plan anual de residentes de Lima, se cumplía en el 48,7% y que la calidad asistencial de los hospitales era percibida como buena en un 63,5%, lo que sustenta los hallazgos sobre las necesidades de formación cubiertas por la sede del residente de cardiología.

Sobre rotaciones externas que reforzarán su formación y la afectación de la misma por la pandemia, nuestros hallazgos son compatibles con los estudios de Reaño y Balderrama, quienes encontraron que más del 60,0% de los residentes manifestaron que su formación se vio afectada por la pandemia de la COVID-19 ^23^^,^^24^, indicando que la imposibilidad de realizar rotaciones, disminución del número de procedimientos y la reducción de las prestaciones hospitalarias y ambulatorias fueron los principales motivos ^13^^,^^18^.

Entre los residentes que cumplieron sus cursos, casi la totalidad indicó que desarrolló los cursos previstos en su plan, aunque en la mitad no fueron desarrollados puntualmente y en 3 de cada 4 se completaron los contenidos del curso. Los medios mediante los cuales se impartieron los contenidos fueron variados, prevaleciendo los medios virtuales, principalmente vídeos a través de internet, difundidos de manera sincrónica y asincrónica. Como se dio en otros escenarios, casi la totalidad se realizó utilizando la virtualidad, mediante el uso de videoconferencias, en su mayoría sincrónicas. Esta modalidad de formación para residentes, también ha sido implementada en otras especialidades y países, como lo muestran diversos estudios ^23^^,^^25^^,^^26^.

En los aspectos relacionados con la salud del residente, específicamente la salud mental y la prevención de enfermedades infecciosas, procesos los cuales están definidos para la acreditación de las sedes docentes y de campos clínicos ^4^^,^^6^, los resultados muestran que las universidades participaron poco en la capacitación de los residentes sobre la COVID-19 donde solo 1 de cada 8 residentes fue capacitado al respecto. En 3 de cada 4 residentes no fue requerido sobre su estado de vacunación y similar proporción sobre el seguimiento o indagación de su estado de salud por parte de la universidad; además, 4 de cada 5 no recibieron actividades orientadas a la preservación de la salud mental del residente durante la pandemia. Estas actividades debieron ser aplicadas considerando que una pandemia influye sobre la salud mental del residente, así como a un riesgo incrementado de infección ^27^^-^^29^.

Finalmente, al explorar la presencia de asociaciones de tipo organizacional, no se evidenció patrón significativo alguno, solo la capacitación sobre aspectos preventivos de la COVID-19 resultó estadísticamente significativa con relación a la universidad privada que la desarrolló para mayor proporción de sus residentes, aunque la proporción fue baja para ambos tipos de universidades. De esta forma, resultaron significativas las asociaciones entre la completa estructuración de los cursos y la proporción de clases desarrolladas de manera sincrónica, a favor de la universidad pública en el primer caso y de la privada en el segundo, pero estas características no están asociadas al nivel de soporte o apoyo en el ámbito asistencial, que es donde los residentes han percibido falencias por parte del apoyo o soporte universitario.

Este último aspecto establece la necesidad de seguir estudiando los niveles de apoyo y soporte formativo que las universidades desarrollan en las sedes docentes, pues si la pandemia ha condicionado decrementos en los niveles de apoyo formativo, también se ha documentado niveles subóptimos de interacción en estudios previos a la pandemia ^21^^,^^22^^,^^30^.

En cuanto a las limitaciones del estudio, se debe tener en cuenta el tamaño muestral, la falta de un proceso de validación formal de los instrumentos empleados y la falta de ajuste de las estimaciones debido al tamaño muestral del estudio.

En conclusión, los resultados de este estudio muestran que la pandemia afectó la interacción entre las universidades, la dinámica prestacional y la formación de los residentes de cardiología de la ciudad de Lima, Perú. Las carencias identificadas en el apoyo a los procesos de formación, en lo relativo a la supervisión y cumplimiento de las rotaciones y el resultado controversial sobre el desarrollo de los cursos de los programas, ha influido negativamente en el desarrollo de la formación de los residentes de cardiología. La formación de este grupo de especialistas no ha alcanzado los niveles y condiciones definidas en los planes curriculares ni de los estándares y perfiles definidos para la especialidad, lo que es claramente percibido por los residentes, que en su mayor proporción han manifestado que su formación ha sido afectada por la pandemia de la COVID-19. Con base en estos hallazgos, es recomendable estudiar si esta estas falencias se modifican positivamente en un contexto posterior al de la pandemia, siendo esta sugerencia basada en que no se evidenciaron diferencias significativas entre el tipo de universidad, indicando que los problemas identificados no solo corresponden a la actuación de la institución formadora, sino también a otras condiciones asociadas por el sistema sanitario. Finalmente, estos resultados deben orientar e impulsar que las autoridades, organizaciones académicas e instituciones sanitarias del país que integran el SINAREME, promuevan iniciativas de evaluación e investigación de los procesos de formación, a fin de actualizar y fortalecer las existentes en la formación de especialistas.
